# Non-surgical endodontic management for the medically complex - hints and tips for the general dental practitioner

**DOI:** 10.1038/s41415-025-8561-2

**Published:** 2025-04-11

**Authors:** Kathryn Finn, Dariusz Kasperek, Sanaa Aljamani, Charlotte Wilson-Dewhurst

**Affiliations:** 41415430384001 Specialist Dentist, Special Care Dentistry, Special Care Dentistry Department, Liverpool University Dental Hospital, Pembroke Place, Liverpool, L3 5PS, UK; 41415430384002 Academic Clinical Fellow in Endodontics, Restorative Dentistry Department, Liverpool University Dental Hospital, Pembroke Place, Liverpool, L3 5PS, UK; 41415430384003 Department of Restorative Dentistry, School of Dentistry, University of Jordan, Queen Rania Street, Amman 11942, Jordan; 41415430384004 Consultant in Special Care Dentistry, Special Care Dentistry Department, Liverpool University Dental Hospital, Pembroke Place, Liverpool, L3 5PS, UK

## Abstract

The management of patients with additional medical complexities is challenging and can cause apprehension when considering if dental treatment is able to be provided in primary care. Non-surgical endodontic treatment is generally a low-risk procedure for many patient cohorts. This paper describes commonly seen medical conditions, the impact on bleeding and infection, and where extraction would preferably be avoided. It explores the risk assessment of these conditions and provides ‘hints and tips' for the provision of endodontic treatment, allowing for the safe and effective provision of dental care, in the appropriate setting, by the appropriate specialty.

## Key points

- Emphasises the role of multidisciplinary collaboration in providing safe and effective care for medically complex patients.
- Explores the essential adaptations required in endodontic treatment for patients with complex medical histories.
- Offers practical endodontic tips tailored for general dental practitioners.

## Introduction

Due to the ageing United Kingdom (UK) population and advances in modern medicine, increasing numbers of patients have medical complexities that have the potential to impact on the delivery of their dental care.^1^

Tooth retention is also increasing in this population, resulting in a rising demand and need for endodontic treatment, placing pressure on both primary and secondary care services.^2^ For most patients, successful endodontic treatment can be completed in primary care, while observing the continually renewed endodontic guidance regarding case complexity and onward referral.^3^^,^^4^ From a medical perspective, it is essential to effectively evaluate the severity and stability of a patient's systemic condition(s) to determine the adaptations to care that are required and when onward referral is necessary. This ensures that unnecessary referral, which can delay a patient's access to care and increase waiting times for those requiring specialist input, is avoided.

The first part of this paper aims to describe medical conditions where the provision of endodontic treatment is complicated by a medical risk, bleeding, or infection, or in instances where avoiding extraction is indicated. It aims to provide the reader with the skills to appropriately risk-assess the patient and offer safe endodontic care and help develop an understanding of how medical conditions can influence whether or not endodontic treatment is preferred to exodontia.

The second part of the paper aims to present endodontic techniques and adaptations which can be implemented to reduce the risks and challenges experienced by this patient cohort.

## Bleeding conditions

There are several bleeding disorders that may have an impact on the endodontic management of a patient with a wide range of aetiologies (Table 1). The more common conditions will be covered within the scope of this paper; however, the principles of providing treatment are applicable to all.

**Table 1:** Summary of bleeding disorders and their aetiologies^7^

**Aetiology**	**Bleeding disorders**
Coagulation factor deficiencies	Congenital: haemophilia A, haemophilia B (Christmas disease) and Von Willebrand disease Acquired: anticoagulants, liver disease, disseminated intravascular coagulation, vitamin K deficiency
Vascular defects	Congenital: connective tissue disease, (Ehlers-Danlos syndrome), Osler-Weber-Rendu syndrome Acquired: senile purpura, infections, steroids, scurvy
Decreased marrow production	Aplastic anaemia, marrow infiltration (leukaemia, myeloma), marrow suppression (cytotoxic drugs, radiotherapy)
Excessive platelet destruction	Immune thrombocytopenic purpura, systemic lupus erythematosus, chronic lymphocytic leukaemia, heparin treatment, viruses, thrombotic thrombocytopenic purpura, sequestration (as in hypersplenism)
Platelet defects	Myeloproliferative disease, increase urea, Von Willebrand disease, Bernard-Soulier (giant platelet) syndrome, alcoholism, drug-induced (aspirin, non-steroidal anti-inflammatory drug)

Dental procedures which pose a risk of bleeding include extractions, incision and drainage of swellings, full periodontal examination, subgingival professional mechanical plaque removal and restorations with subgingival margins.^5^ Local anaesthetic techniques also need to be considered, as administration of inferior alveolar nerve blocks and lingual infiltrations can pose the risk of haematoma in those with the most severe bleeding diathesis, placing them at risk of trismus and airway compromise.^6^

### Factor deficiencies

Bleeding disorders affect around one in 2,000 people in the UK, with the three most common congenital disorders being von Willebrand disease (1% of UK population when including all forms, with the prevalence of symptomatic disease requiring treatment being around 1 in 10,000 people), haemophilia A (25 cases per 100,000 male births) and haemophilia B (five cases per 100,000 male births).^8^ They range in their severity and management and often require liaison with the haematology team as to what dental treatment is appropriate in the primary care setting. Therefore, it is good practice to consult the patient's haematologist before delivering any invasive care to ascertain if any precautionary measures need to be taken.

Depending on the dental treatment indicated, patients may require a range of haematological preparations before attending. These preparations include tranexamic acid (topical and oral), desmopressin and factor replacement, and are dictated by the patient's condition.^8^^,^^9^ Some preparations may need to be directly administered by the haematology team, which necessitates close liaison when organising the timing of appointments. It is therefore often more practical for these patients to be treated in a setting close to a haematology centre. Other preparations, such as oral tranexamic acid and the immunological medication emicizumab, are self-administered by the patient. The introduction of emicizumab for the management of haemophilia A, and increasing numbers of patients being responsible for providing their own haematological preparation, have reduced the barriers for these patients accessing some dental treatment in primary care.^10^

From an endodontic perspective, the World Federation of Hemophilia guidance states that non-surgical endodontic management is generally a low-risk procedure in terms of bleeding.^11^ However, consideration must be given to the preferred local anaesthetic technique, as administering an inferior dental block (IDB) is considered a higher-risk procedure for bleeding.^11^

To minimise bleeding risk, buccal infiltrations with articaine or intraosseous or intraligamentary anaesthesia could be used as an alternative to an IDB.

### Platelet disorders

As with factor deficiencies, non-surgical endodontic management is considered a low-risk procedure. The minimum recommended platelet count required for endodontic treatment varies between haematology centres. When providing endodontic care, the haematology team must be consulted for all patients with a platelet count of less than 50 × 10^9^. ^12^^,^^13^ In patients with platelet defects (Table 1), platelet levels may be normal or even raised. Liaison with the haematology team is essential to determine if haematological management is required, as qualitative defects in platelets may significantly impair clotting.

## Cardiology conditions

Heart and circulatory diseases affect more than 7.6 million people in the UK and subsequently will be regularly encountered by a general dental practitioner. Cardiovascular diseases encompass a wide range of conditions, including ischemic heart disease (IHD), heart failure, cardiac arrhythmias and infective endocarditis (IE).

### Assessing the stability and severity

Assessing the severity and stability of a cardiac condition is essential to determine if endodontic treatment can be provided safely in a primary care setting. Angina is a common clinical feature of IHD, which presents typically as chest pain resulting from myocardial ischaemia during periods of increased exertion. Angina episodes increasing in frequency, new onset of severe angina, pain occurring at rest, and prolonged episodes of pain lasting longer than 15-20 minutes, are all indications of unstable angina.^14^

Additionally, patients with IHD are at risk of myocardial infarction (MI). Historic guidelines state that elective dental care should be avoided in the six months immediately post-MI, on the basis that the risk of complications is highest during this period.^15^ However, emerging evidence suggests the period in which elective care should be avoided is as little as 4-6 weeks post-MI.^16^ Patients requiring urgent management of dental pain who have recently had an MI should be referred onwards for treatment in a hospital setting where facilities to manage a potential medical emergency are readily available.^17^

Cardiac arrhythmias range from mild conditions which have little to no effect on cardiac function, to life threatening arrhythmias which require immediate medical management. The presence of symptoms, such as fatigue, dizziness, syncope and angina, are all indications of a poorly controlled arrhythmia.^18^ In addition, asking a patient how regularly they see a cardiologist provides insight into both the severity and stability of the condition.

Heart failure is a progressive disease which has a minimal impact on endodontic treatment in the early stages, where numerous compensatory mechanisms maintain cardiac output. As the disease progresses, cardiac function deteriorates and pulmonary congestion results, leading to dyspnoea. Subsequently, patients may struggle to lie supine. Asking a patient about the position they sleep in can help guide whether they are likely to tolerate treatment in a supine position. Endodontic treatment may be contraindicated if there is insufficient access due to patient positioning.

In patients with unstable heart disease, which poses a constant threat to life and significant risk of a medical emergency occurring, further medical or surgical management may be planned, in which case, elective endodontic care should be delayed until the disease is more stable.^19^^,^^20^ In instances where treatment cannot be delayed or the stability is unlikely to improve, patients should be treated in a specialist setting in close liaison with the cardiologist.^20^ For example. for patients with unstable cardiac conditions, treatment in a hospital or theatre setting in the presence of, or following consultation with, an anaesthetist may be required.

### Pain and anxiety management

Pain and anxiety can increase heart rate and risk of angina, or induce arrhythmias, so excellent pain and anxiety management is essential.^18^^,^^21^

In patients with known arrhythmias, the use of large volumes of local anaesthetic with adrenaline should be avoided.^18^ However, it is imperative that analgesia is adequate during endodontic therapy to avoid pain, stress and anxiety, and the resultant release of excessive endogenous catecholamines.^22^ Thus, local anaesthetic with adrenaline should not necessarily be avoided, and evidence suggests that using less than four cartridges of lidocaine with adrenaline 1:80,000 was relatively safe for cardiovascularly compromised patients.^23^

Alternative treatment modalities, such as conscious sedation, should be considered where patients have significant dental anxiety to reduce the stress experienced. Inhalation sedation with nitrous oxide is of particular benefit for those with cardiovascular conditions due to additional availability of oxygen and the beneficial effect of increasing cardiac output.^24^

### Anticoagulation

Patients with heart disease may be anticoagulated. Anticoagulants include anti-platelet medications, such as aspirin and clopidogrel; direct oral anticoagulants; low-molecular-weight heparins, such as dalteparin and enoxaparin; and the vitamin K antagonist warfarin. As previously discussed, non-surgical endodontics is a low-risk bleeding procedure and therefore can be safely carried out in these patients. Both infiltration anaesthesia and IDBs can be safely administered; however, if an IDB is required for a warfarinised patient, the international normalised ratio (INR) should be below 4.0. The INR should be measured no more than 72 hours before the procedure and no more than 24 hours before for those where the INR is unstable. Excellent guidelines published by the Scottish Dental Clinical Effectiveness Programme (SDCEP) on the management of patients taking anticoagulant or antiplatelet drugs are available and the reader is encouraged to read these for further information.^5^

### Risk of infective endocarditis

IE is a rare, life-threatening infection affecting the endocardium; in particular, the heart valves. SDCEP provide implementation advice for the National Institute for Health and Care Excellence (NICE) antibiotic prophylaxis guidelines published in 2015, providing guidance as to how to manage patients presenting with an increased risk of IE.^25^^,^^26^ It is worth noting that the European Society of Cardiology guidelines differ in terms of when antibiotic prophylaxis is recommended, and the prescribing regimen and NICE are currently reviewing their guidelines.^26^^,^^27^ The reader should therefore ensure that they refer to the most up-to-date guidance.

Patients at increased risk of IE can be seen in Table 2. Patients who require special consideration are at particularly high risk of contracting IE with serious and potentially life-threatening consequences.^25^^,^^28^

**Table 2:** Patients at risk of infective endocarditis*

**At risk of IE**	**Requiring special consideration**
Acquired valvular heart disease (stenosis/regurgitation) Hypertrophic cardiomyopathy Previous IE Structural congenital heart disease (including those that have been repaired)** Valve replacement	Prosthetic valve (where any prosthetic material has been used for repair) Previous IE Any type of congenital heart disease, including those repaired with a prosthetic material
Key: * = Adapted from SDCEP's antibiotic prophylaxis advice^25^ ** = Excluding isolated atrial septal defect, fully repaired ventricular septal defect, fully repaired patent ductus arteriosus, and closures that have epithelialised	

Most patients can receive their dental treatment routinely and should be strongly encouraged to maintain good oral hygiene. It is important to discuss the risk of IE, highlighting the main symptoms of infection and the risks and benefits of antibiotic prophylaxis.^25^

For those patients considered to be of special consideration, in addition to the discussions listed above, liaison with the patient's cardiologist is necessary to agree if antibiotic prophylaxis is required.^25^

Although available evidence is conflicting, it has been suggested that placement of a subgingival matrix band and endodontic treatment where an apical stop has not been established are considered high-risk procedures for those with special considerations.^25^^,^^27^^,^^28^ Therefore, most endodontic treatments require a liaison with the patient's cardiologist and possible administration of antibiotics before the patient's procedure.

### Dental assessment before surgery

Dental assessment before cardiac surgery is recommended to remove any source of infective foci that could risk the outcome of the cardiac procedure, or cause pain and infection during the patient's hospital stay.^29^

The patient's medical and cardiac stability would dictate the setting in which the patient receives the assessment and any appropriate treatment. This can be completed in liaison with the patient's cardiology team.^29^

The timeframe from assessment to surgery is usually short and therefore the urgency and predictability of treatment needs to be considered.^29^ Prioritisation is often focused on extraction of unrestorable/infected teeth and optimising the patient's oral hygiene.^29^^,^^30^ Endodontic treatment is often a procedure requiring multiple appointments and resolution of the apical infection may not be possible in the timeframe available, as it risks placing the patient at risk of pain and infection during their surgical stay. If time allows the prioritisation of the endodontic management, then the patient's medical stability, the setting and the experience of the operator, should be considered, to increase the predictability of a successful endodontic outcome.

## Oncology management

The cancer process and its management can impact on the provision of dental care, with many resultant side effects that affect the oral cavity. The common cancer therapies include chemotherapy, immunotherapy, radiotherapy and haematopoietic stem cell transplant.^31^^,^^32^ Many of the cancer therapies result in pancytopenia; those of particular importance to dental treatment would be platelet and neutrophil levels, as this would increase their risk of infection and risk of bleeding during invasive dental treatment.^33^ Other oral complications of this patient cohort include mucositis, xerostomia and trismus, which can impact on the patient's ability to maintain oral hygiene and contribute to an increased risk of caries and periodontal disease.^32^^,^^33^

It is recommended that patients receive a dental assessment and any necessary dental treatment before commencing cancer therapy.^33^^,^^34^^,^^35^ The assessment aims to remove any source of infection that could impact on the patient's oncology management.^33^ Most patients on this pathway are likely to be referred to a specialist centre for completion of this treatment; however, there are areas of the country where this pathway has not been established.

Although an early pre-treatment assessment is recommended, the timeframe available prior to the patient receiving their care is often limited.^33^ Therefore, a pragmatic and predictable focus on treatment planning, including extraction of teeth of poor prognosis and stabilisation of restorable teeth, is required.

Providing endodontic treatment is not necessarily contraindicated for this patient cohort. However, the number of appointments and time available to allow for predictable completion, along with resolution of symptoms (pain and infection), need to be considered, thus reducing the risk of the patient experiencing symptoms during their cancer treatment, where access to dental care is limited or impossible.

The patient's medical stability and stage of cancer therapy must be considered in relation to the provision of dental care. Although endodontic treatment is considered a low-risk procedure, liaison with the oncology team is essential to establish at what stage in the cancer management the endodontic treatment should occur and whether the patient would require any haematological support before the appointment.

### Relevant side effects of oncology management

#### Medication-related osteonecrosis of the jaw

There are many medications used in oncology treatment which are implicated in the development of medication-related osteonecrosis of the jaw (MRONJ). The risk of developing MRONJ post-extraction has been reported as between 0-18% for patients treated with intravenous (IV) bisphosphonates for cancer.^36^ As bisphosphonates irreversibly bind to bone, this risk persists even following cessation of the drug. Denosumab, an antiresorptive drug, is also implicated and is used in the treatment of metastatic bone disease. As for IV bisphosphonates, most studies demonstrated a risk of less than 5% for oncology patients.^36^ Due to its different mechanism of action, the risk of MRONJ in patients taking denosumab decreases following cessation of the drug and current evidence suggests that the risk is low in patients who have not taken it in over nine months.^37^ It is beyond the scope of this paper to discuss all the drugs that present a risk of MRONJ and readers are directed to the SDCEP guidelines for further guidance.^38^

#### Osteoradionecrosis

Osteoradionecrosis (ORN) presents as an area of exposed necrotic bone or a fistula in an area of the mandible or maxilla which has been exposed to radiotherapy. Osteoradionecrosis is found more commonly to affect the mandible but can present in all areas of the mouth.^39^ The dose of radiation also has a bearing on the risk with doses of 45 Gray or above quoted as the threshold for ORN development.^33^ In considering dental management, it is important to consult with a patient's oncology team to ascertain both the field of radiotherapy and the dose to help determine whether ORN is a risk.

#### Treatment planning

ORN and MRONJ can both lead to significant long-term morbidity. The most implicated risk factor for their development is the extraction of teeth; thus, endodontics can play a key role in the prevention of these side effects.^33^

Therefore, it may be appropriate to consider root-treating retained roots and teeth that aren't restorable by conventional means to avoid extraction. However, the key principles of endodontics remain and a coronal seal, in some form, should be provided to avoid subsequent endodontic failure and infection.^40^ This is of particular importance, as periapical infection itself is a risk factor for the development of both MRONJ and ORN.^41^^,^^42^

Trismus may complicate endodontic treatment in patients who have undergone treatment for head and neck cancer because of both surgical management and radiotherapy. Access for treatment may be further compromised by altered anatomy following surgical excision and reconstruction. In addition, dysphagia presents a risk in this patient group, with some patients' swallowing permanently impaired, placing them at risk of aspiration pneumonia.^43^^,^^44^ Careful assessment is therefore required before undertaking endodontic care, as the use of a high-speed handpiece and ultrasonic instruments may be completely contraindicated in those with severe dysphagia if perfect isolation with dental dam cannot be achieved.

## Endodontic management

Regardless of medical complexities, the restorability of the tooth and optimum coronal seal remain paramount in determining the predictability of endodontic success.^45^ Table 3 highlights, from the medical conditions discussed, the endodontic considerations that should be undertaken to allow for successful and safe provision of endodontic care.

**Table 3:** Endodontic adaptations for managing medically complex patients

**Medical consideration**	**Endodontic adaptation**	**Why?**
Bleeding disorders	Rubber dam isolation	Reduce trauma to the gingivae and minimise bleeding
	Single-visit endodontics	Reduce the number of appointments where haematological preparation is required
	Working length considerations	Reduce over-instrumentation of the apex and thus risk of bleeding and bacteraemia
Cardiology conditions	Single-visit endodontics	Reduce the number of visits requiring antibiotic prophylaxis and thus reduce the risk of antimicrobial resistance
Oncology management	Rubber dam isolation	Reduce risk of aspiration, especially in patients with compromised cough reflex or dysphagia Minimise risk of soft tissue trauma, including gingival injury and mucosal irritation Allow for isolation of roots/teeth with minimal coronal structure
	Single-visit endodontics	Minimise the number of appointments and completion of treatment in a timeframe
	Managing limited mouth opening	Trismus is a recognised side effect of radiotherapy treatment
	Working length considerations	In patients with a risk of bleeding, reduce over-instrumentation of the apex Reduce the risk of extrusion of debris, minimising flare-ups and postoperative infections
	Restorability of tooth and coronal seal	To avoid extractions (ORN/MRONJ risk), teeth may require adaptation to allow placement of coronal restoration

### Rubber dam isolation

**Fig. 1:**
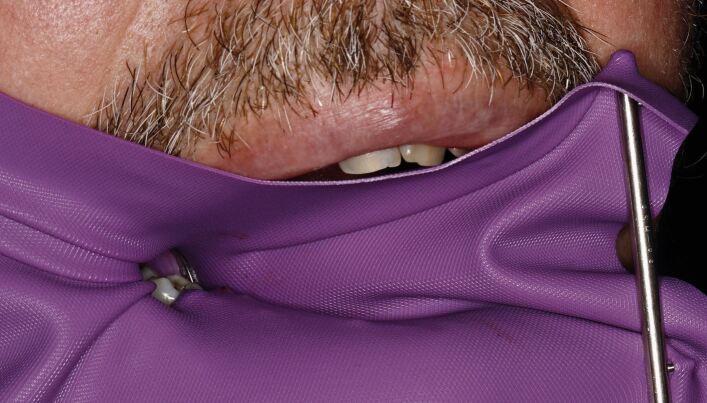
Lowered rubber dam placement creating a breathing space for a patient with an obstructed nasal airway while maintaining isolation

The use of a rubber dam (RD) is critical for maintaining a dry, clean, operative field during endodontic treatment. In cases of heavily broken-down teeth or retained roots, the split dam technique, combined with the use of a flowable liquid dam, can be employed to achieve effective isolation. To further minimise trauma to the gingival tissues, a careful selection of rubber dam application is essential. Polymer non-traumatic RD clamps, such as SoftClamp (Kerr, Switzerland), or use of Wedjets elastic stabilising cord, should be considered to prevent unnecessary soft tissue trauma. Additionally, floss ties or composite resin tags can be placed on the tooth surface to ensure optimal rubber dam fit and security. For patients with nasal breathing difficulties, such as those with an obstructed airway, adjusting the rubber dam placement can offer significant relief. Lowering the rubber dam frame slightly or creating a small breathing window at the base of the dam can help maintain adequate ventilation without compromising the isolation of the treatment site (Fig. 1).

Although sodium hypochlorite remains the gold-standard irrigant for endodontic treatment due to its effective antimicrobial properties, other irrigants, such as chlorhexidine, can be considered in cases where achieving an effective coronal seal is challenging. Chlorhexidine has a broad spectrum of antimicrobial activity against both Gram-positive and Gram-negative bacteria, as well as antifungal efficacy against *Candida albicans*; however, its effect on microbial biofilms is significantly weaker than sodium hypochlorite and it has minimal tissue solubility.^46^ This may be advantageous in situations where chemical properties of sodium hypochlorite pose patient safety concerns due to the risk of leakage.

### Single-visit versus multiple-visit endodontics

Single-visit endodontics should be strongly considered in medically complex patients due to its advantages, including reduced risk of bleeding/trauma episodes and subsequently the reduced need for factor replacement and other haematological adjuncts, which can be costly and have side effects. In addition, there is greater convenience for both patients and carers, and decreased anxiety associated with multiple visits, all while maintaining the same reported level of effectiveness as multi-visit treatment.^47^ The single-visit approach is particularly recommended for patients with asymptomatic apical periodontitis, as supported by the latest European Society of Endodontology S3 guidance.^4^ Reciprocating file systems, such as WaveOne Gold (Dentsply Maillefer, Switzerland) or Reciproc Blue (VDW, Munich, Germany), can be especially useful due to a simple file sequence and minimal number of files required for the shaping of the root canal system.

### Managing limited mouth opening

**Fig. 2:**
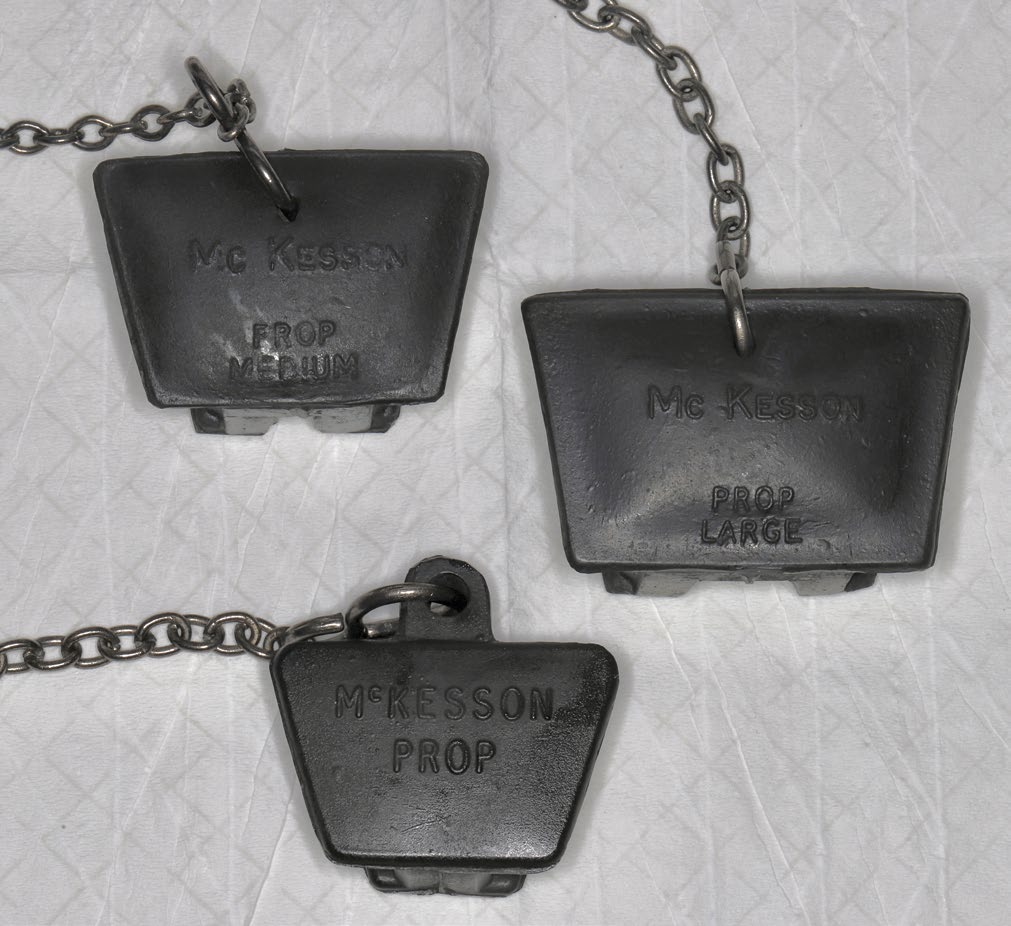
McKesson mouth props (BR Surgical, Germany)

For patients with trismus, using mouth props can provide a comfortable and stable working space. This enhances access to the treatment site for the dentist while providing patient comfort (Fig. 2).

**Fig. 3:**
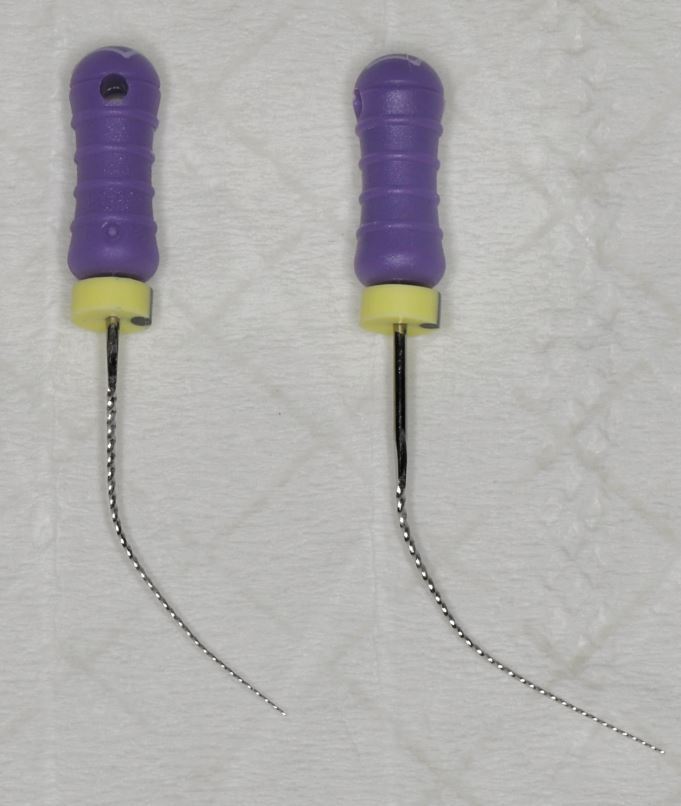
21 mm (left) vs 25 mm (right) K file

**Fig. 4:**
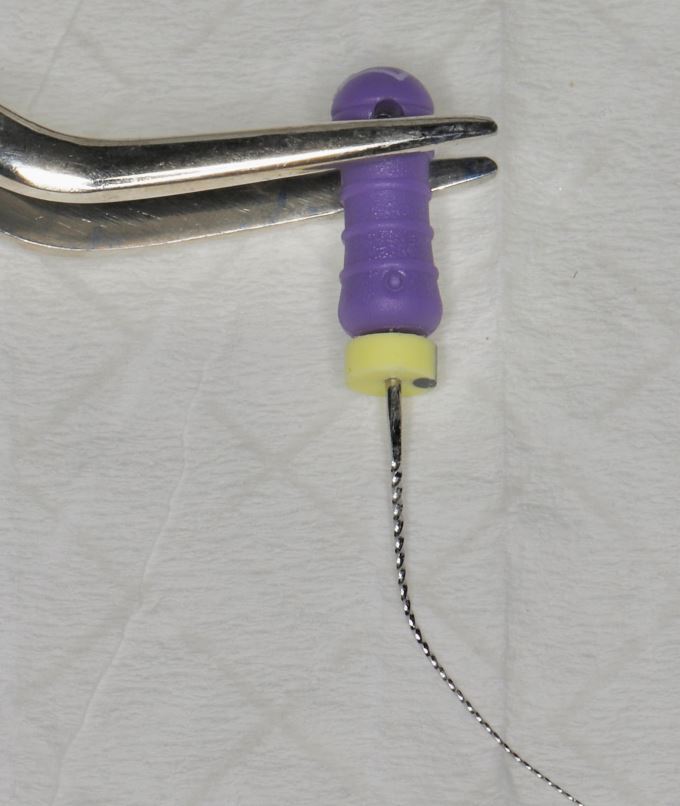
Use of tweezers with a pre-bent K file

**Fig. 5:**
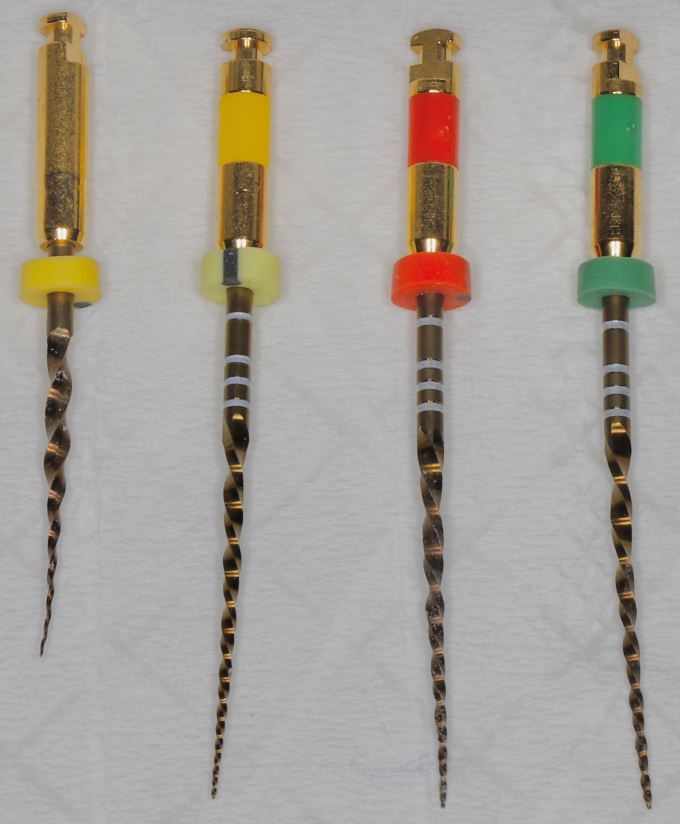
Comparison of length of orifice opener file ProTaper Gold (19 mm) (left) against 25 mm WaveOne Gold family of files

Furthermore, use of shorter endodontic files (21 mm length instead of standard 25 mm) is recommended (Fig. 3), as they allow better manoeuvrability and access within the restricted working space. Pre-bending of the endodontic file and use of tweezers can be used to guide the endodontic instruments precisely into the canal orifices, reducing the need for excessive hand movement and improving visualisation (Fig. 4). In the initial stages of root canal instrumentation, orifice openers, such as the ProTaper Gold SX file (Dentsply Maillefer, Switzerland) can also be used. These shorter-length files facilitate access and shaping of the coronal portion of the canal, which reduces the stress on the subsequent instrumentation files and provides straight-line access (Fig. 5).

**Fig. 6:**
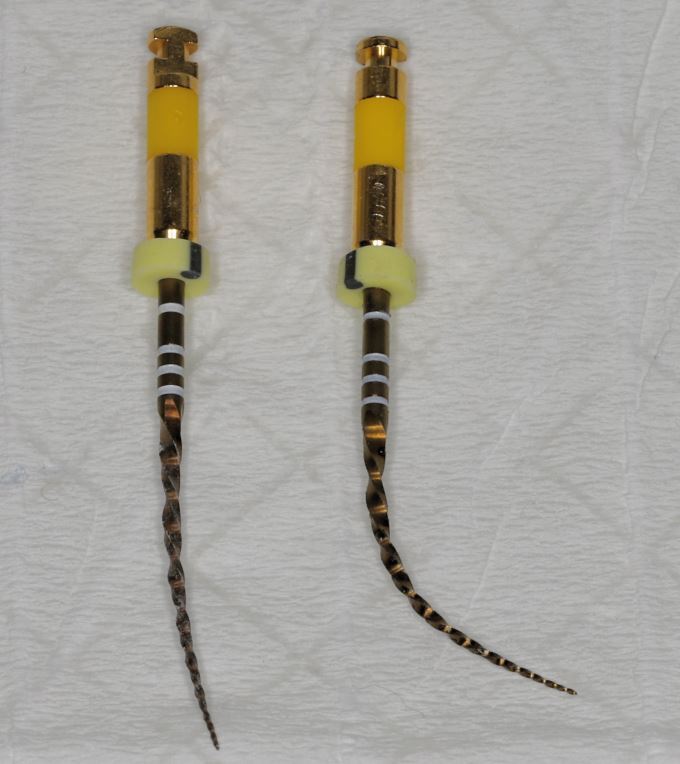
WaveOne Gold small file, before (left) and after (right) application of refrigerant spray

Additionally, refrigerant spray, such as Endofrost (Roeko, Germany), can be used to cool and temporarily modify the properties of nickel-titanium endodontic instruments, enabling greater flexibility in the manipulation to reach root canal orifices (Fig. 6). Using martensitic file systems, such as HyFlex EDM (Coltene, Switzerland) can also be beneficial in such cases, as they offer enhanced flexibility.

### Working length considerations

Establishing the correct working length is crucial to protect the apical anatomy and minimise the risk of over-instrumentation beyond the apex, which can increase the potential for bleeding. This is especially important in the management of curved canals, where the canal curvature tends to straighten following shaping, leading to a decrease in the overall working length; therefore, frequent use of the electronic apex locator (EAL) is recommended. EALs are more accurate than using periapical radiographs alone.^48^ Additionally, the use of the periapical radiographs should be minimised for the bleeding patient cohort due to the low risk associated with intra-oral trauma during film placement.^49^

In the case of suspected prolonged apical bleeding, it is essential to confirm that all pulpal tissue has been fully removed from the apical and lateral portions of the root canal system through instrumentation and copious irrigation with sodium hypochlorite. Paper points soaked in a refrigerant spray such as Endofrost (Roeko, Germany), together with application of gentle apical pressure, can be used to effectively manage a bleeding apex. However, caution should be exercised when using refrigerant sprays, as excessive use may cause localised tissue necrosis. Additionally, injecting a local anaesthetic solution with adrenaline into the canal space or applying adrenaline topically (in a 1:1,000 concentration) using paper points can be effective in reducing bleeding.^49^ If apical bleeding cannot be controlled due to factors such as severe inflammation or infection, placing an intracanal medicament and scheduling a follow-up visit may be appropriate.

### Restorability of the tooth and obturation

Single cone obturation with a hydraulic calcium-silicate cement (HCSC) sealer is a suitable approach for this patient cohort, as it provides a simplified technique that minimises procedural time while maintaining an effective seal.^50^ HCSCs have multiple desirable properties, including biocompatibility and excellent sealing abilities.^51^ In cases where teeth are deemed unrestorable and extraction is contraindicated due to the risk of developing ORN or MRONJ, an alternative obturation with sole use of HCSCs can be performed. This approach can also be used in endodontic retreatments and in teeth with unusual anatomy.^52^

The restorability of the tooth and ability to maintain adequate coronal seal plays an important role in determining the success of endodontic treatment.^45^ It is essential to ensure that the treated teeth can be adequately restored with an effective coronal seal. To achieve this, a minimum of 3 mm of restorative material, such as resin-modified glass ionomer cement or flowable composite, should be placed as an orifice seal, with the access cavity subsequently restored using conventional resin composite.^53^ In previously restored posterior teeth, where one or more marginal ridges have been lost, cuspal protection should also be considered.^53^

## Conclusion

The endodontic considerations discussed in this paper can be applied across a range of medical conditions where adaptation of dental management is necessary. It must be noted that dental guidance is continually reviewed and healthcare professionals should be aware when changes are implemented. Successful endodontic treatment in medically complex patients relies upon selecting the most appropriate treatment modality, careful assessment of the severity and stability of the patient's medical condition, and ensuring treatment is provided in the correct setting by clinicians with the relevant expertise. Maintaining optimal oral hygiene and achieving an adequate coronal seal remain critical factors in determining long-term endodontic success.
