# Correction to: Non-surgical endodontic management for the medically complex – hints and tips for the general dental practitioner

**DOI:** 10.1038/s41415-025-8766-4

**Published:** 2025-05-23

**Authors:** Kathryn Finn, Dariusz Kasperek, Sanaa Aljamani, Charlotte Wilson-Dewhurst

**Affiliations:** 791092535489628032765 Specialist Dentist, Special Care Dentistry, Special Care Dentistry Department, Liverpool University Dental Hospital, Pembroke Place, Liverpool, L3 5PS, United Kingdom; 337408735912734239576 Academic Clinical Fellow in Endodontics, Restorative Dentistry Department, Liverpool University Dental Hospital, Pembroke Place, Liverpool, L3 5PS, United Kingdom; 916941151452644376752 Department of Restorative Dentistry, School of Dentistry, University of Jordan, Queen Rania Street, Amman 11942, Jordan; 161576079453165731184 Consultant in Special Care Dentistry, Special Care Dentistry Department, Liverpool University Dental Hospital, Pembroke Place, Liverpool, L3 5PS, United Kingdom

Author's correction note:

Clinical article *Br Dent J* 2025; **238:** 551–557.

When initially published, there was an error in one of the author's affiliations. The affiliation for Sanaa Aljamani read ‘Consultant in Endodontics, Restorative Department, Jordan University Hospital, Queen Rania Street, Amman 11942, Jordan' but should have read ‘Department of Restorative Dentistry, School of Dentistry, University of Jordan, Queen Rania Street, Amman 11942, Jordan'.

The authors apologise for any inconvenience caused.

